# The estimated GFR, but not the stage of diabetic nephropathy graded by the urinary albumin excretion, is associated with the carotid intima-media thickness in patients with type 2 diabetes mellitus: a cross-sectional study

**DOI:** 10.1186/1475-2840-9-18

**Published:** 2010-05-15

**Authors:** Hiroyuki Ito, Yuko Komatsu, Mizuo Mifune, Shinichi Antoku, Hidenori Ishida, Yuichiro Takeuchi, Michiko Togane

**Affiliations:** 1 Department of Diabetes, Metabolism and Kidney Disease, Edogawa Hospital, Tokyo, Japan; 2 Department of Laboratory, Edogawa Hospital, Tokyo, Japan

## Abstract

**Background:**

To study the relationship between the intima-media thickness (IMT) of the carotid artery and the stage of chronic kidney disease (CKD) based on the estimated glomerular filtration rate (eGFR) and diabetic nephropathy graded by the urinary albumin excretion (UAE) in the patients with type 2 diabetes mellitus.

**Methods:**

A cross-sectional study was performed in 338 patients with type 2 diabetes mellitus. The carotid IMT was measured using an ultrasonographic examination.

**Results:**

The mean carotid IMT was 1.06 ± 0.27 mm, and 42% of the subjects showed IMT thickening (≥ 1.1 mm). Cerebrovascular disease and coronary heart disease were frequent in the patients with IMT thickening. The carotid IMT elevated significantly with the stage progression of CKD (0.87 ± 0.19 mm in stage 1, 1.02 ± 0.26 mm in stage 2, 1.11 ± 0.26 mm in stage 3, and 1.11 ± 0.27 mm in stage 4+5). However, the IMT was not significantly different among the various stages of diabetic nephropathy. The IMT was significantly greater in the diabetic patients with hypertension compared to those without hypertension. The IMT positively correlated with the age, the duration of diabetes mellitus, and the brachial-ankle pulse wave velocities (baPWV), and negatively correlated with the eGFR. In a stepwise multivariate regression analysis, the eGFR and the baPWV were independently associated with the carotid IMT.

**Conclusions:**

Our study is the first report showing a relationship between the carotid IMT and the renal parameters including eGFR and the stages of diabetic nephropathy with a confirmed association between the IMT and diabetic macroangiopathy. Our study further confirms the importance of intensive examinations for the early detection of atherosclerosis and positive treatments for hypertension, dyslipidaemia, obesity, as well as hyperglycaemia are necessary when a reduced eGFR is found in diabetic patients.

## Background

Diabetic nephropathy is a major manifestation of microangiopathy that plays a significant role in the prognosis of patients with diabetes mellitus. An increased number of individuals with end-stage renal failure caused by diabetic nephropathy present a large social problem. Diabetic nephropathy is graded according to the urinary albumin excretion rate (UAE). Microalbuminuria as well as macroalbuminuria are important markers for the progression of renal dysfunction and are currently recognized as predictive factors for cardiovascular adverse events [1-3].

Recently, it has become possible to evaluate the glomerular filtration rate (GFR) using a formula to calculate the estimated GFR (eGFR) in Japanese subjects [4]; although serum creatinine levels and the creatinine clearance rate have long been used for the evaluation of kidney function. Chronic kidney disease (CKD) is defined as the decrease in eGFR (<60 mL/min/1.73 m^2^) for 3 months or more and/or persistent proteinuria [5]. The stages of CKD are based only on the GFR and it is also acknowledged that the risk of a cardiovascular event increases with the progression of the CKD stage.

The measurement of the intima-media thickness (IMT) enables the detection of atherosclerotic lesions of the arterial walls. It is well-known that atherosclerotic diseases frequently occur in diabetic patients as diabetic macroangiopathy. In patients with type 2 diabetes mellitus, the carotid IMT is significantly greater than in the corresponding healthy, age- and sex-matched, non-diabetic subjects [6-9]. A small increase in the IMT of the carotid artery predicts coronary heart disease and stroke even after an adjustment for cardiovascular risk factors [10]. Therefore, the IMT is considered to reflect an early stage of macroangiopathy in diabetic patients. The carotid IMT was used as a surrogate marker of diabetic macroangiopathy in the cohort study because the number of macrovascular events was rather small [11].

Several studies have reported the IMT to increase with the progression of diabetic nephropathy; however, the relationship of the IMT with the UAE is still controversial [12-17]. An association of the IMT with both the UAE and the GFR in diabetic patients has been investigated in a few reports [12,17]. Previous investigations did not observe a relationship between the IMT and the renal parameters after an association between the IMT and diabetic macroangiopathies was reported in a single study.

The aim of this study was to investigate the relationship of the carotid IMT with the clinical backgrounds, including diabetic complications, stages of CKD, and diabetic nephropathy, of Japanese patients with type 2 diabetes mellitus.

## Methods

A cross-sectional study was performed in a population of 338 patients diagnosed with type 2 diabetes mellitus under consecutive evaluations, including urinalysis, serum creatinine levels, and ultrasonographic examinations for the carotid artery at the Department of Diabetes, Metabolism and Kidney Diseases of Edogawa Hospital, Tokyo, Japan between April 2008 and March 2009. The patients with end-stage renal disease receiving maintenance dialysis were excluded from this study because it is more difficult to evaluate clinical parameters such as blood pressure, body weight, serum creatinine and lipid concentrations in these patients compared with those not on dialysis. Subjects who underwent physical health examinations were also entered into the study as normal controls (age-matched 37 men and 30 women). They did not have diabetes mellitus, hypertension, hyperlipidaemia and the history of myocardial and/or cerebral infarction.

The eGFR was calculated using the formula reported by Matsuo et al [4]. This equation originated from the MDRD study group [18] arranged for Japanese individuals, and it is recommended by the Japanese Society of Nephrology: eGFR (mL/min/1.73 m2) = 194 × Scr^-1.094 ^× Age^-0.287 ^× 0.739 (if female). The stages of CKD were based on the NKF K/DOQI clinical practice guidelines [5].

The UAE is presented as the albumin-to-creatinine ratio (ACR; mg/g creatinine). Diabetic nephropathy was staged according to an analysis of a spot urine sample as: DN stage I (normoalbuminuria), ACR < 30 mg/g creatinine; DN stage II (microalbuminuria), 30 ≤ ACR < 300 mg/g creatinine; DN stage III (macroalbuminuria), ACR ≥ 300 mg/g creatinine (or dipstick urinalysis revealed 2+, 3+ or 4+) and eGFR ≥ 30 mL/min/1.73 m^2^; and DN stage IV, ACR ≥ 300 mg/g creatinine (or dipstick urinalysis revealed 2+, 3+ or 4+) and eGFR < 30 mL/min/1.73 m^2^. The individuals who had additional kidney diseases, such as acute renal failure, chronic glomerulonephritis and interstitial nephritis, were excluded from this study.

The blood pressure was measured twice with the subjects in the sitting position after a 5-minute rest. The lower value of the two measurements was used for the study. Hypertension was defined as a systolic blood pressure ≥ 140 mmHg and/or a diastolic blood pressure ≥ 90 mmHg. The participants currently using antihypertensive medications were also classified as positive for hypertension. Hyperlipidaemia was defined by serum concentrations of total cholesterol ≥ 5.7 mmol/L, LDL-cholesterol ≥ 3.6 mmol/L or as patients already being treated with lipid-lowering agents.

Diabetic retinopathy was defined as a simple retinopathy or as more severe conditions, which was judged according to the results of a funduscopic examination performed by expert ophthalmologists. Diabetic neuropathy was diagnosed by the presence of two or more components among clinical symptoms (bilateral spontaneous pain, hypoaesthesia, or paraesthesia of the legs), the absence of ankle tendon reflexes and decreased vibration sensations using a C128 tuning fork. Cerebrovascular disease was diagnosed by the physicians as a history of an ischaemic stroke using brain computed tomography or magnetic resonance imaging. Only the patients with symptoms were classified as having cerebrovascular disease, and cases of silent brain infarction, transient ischaemic attack or brain haemorrhage were excluded from this study. Coronary heart disease was diagnosed based on a previous history of myocardial infarction, angina pectoris, electrocardiogram abnormalities suggesting myocardial ischaemia or interventions after coronary angiographic examination. Peripheral arterial disease was diagnosed by the absence of a pulse in the legs along with ischaemic symptoms, obstructive findings on an ultrasonographic examination of the lower extremities, or ankle-brachial pressure index (ABI) < 0.9.

The ABI and brachial-ankle pulse wave velocities (baPWV) as indicators of atherosclerosis were measured using Form PWV/ABI, BP-203PRE II (Omron Colin Co., Ltd, Bunkyo, Tokyo, Japan).

The IMT of the carotid artery was measured with ultrasonographic examinations by skilled laboratory technicians using Aplio XV ultrasound machine (Toshiba Medical Systems Corp., Ohtawara, Tochigi, Japan). B-mode imaging of the carotid artery was performed with a 7.5 MHz probe. The distal common carotid artery and the carotid bulb were identified by longitudinal scanning in the supine position with the head slightly extended and turned to the oppose direction. The IMT was defined as the distance between the leading edges of the lumen interface and the media-adventitia interface at the far wall. IMT was not measured at the site of the atheromatous plaques. The bilateral common carotid and the internal carotid arteries were assessed by 2 different (anterior-posterior and lateral) longitudinal projections. The representative IMT was expressed as the maximal value of the measurements at any location in the carotid arteries (max IMT). The final value of the IMT was calculated as an average of the max IMT on both sides in this study. Although the carotid IMT increases with age, it does not rise above 1.0 mm in normal Japanese adults. Therefore, the presence of increased IMT (IMT thickening) in the carotid artery has been defined as an IMT of ≥ 1.1 mm [8].

### Statistical analysis

An analysis of variance (ANOVA) and the χ^2 ^test were used for between-group comparisons of the continuous and categorical variables, respectively. Pearson's univariate regression analysis and stepwise multivariate regression analysis by the forward selection method were performed to determine the association of the carotid IMT with the other clinical parameters. Differences with P < 0.05 (two-tailed) were considered to be statistically significant. The statistical software package JMP, version 8.0 (SAS Institute, Cary, NC, USA), was used to perform all of the analyses.

## Results

Table 1 shows the clinical characteristics and the laboratory parameters of the patients. The mean carotid IMT was significantly greater in the study subjects than in the normal controls (1.06 ± 0.27 mm versus 0.94 ± 0.30 mm, *P *= 0.04).

**Table 1:** Clinical characteristics of the patients.

	%/Mean (SD)	Number estimated (%)
Age (years)	65 (11)	338 (100)
Men	64	338 (100)
Duration of diabetes mellitus (years)	11 (10)	254 (75)
Current plus past smoking	57	282 (83)
Current drinker	43	268 (79)
Therapeutic method for diabetes mellitus		338 (100)
Diet only/OHA/Insulin	4/51/44	
Body mass index (kg/m^2^)	24.4 (4.4)	333 (99)
Obesity^#^	36	333 (99)
Hypertension	78	338 (100)
Systolic blood pressure (mmHg)	132 (19)	338 (100)
Diastolic blood pressure (mmHg)	76 (12)	338 (100)
Antihypertensive agents		338 (100)
ACEi	19	
ARB	46	
CCB	48	
Hyperlipidaemia	73	338 (100)
Statin	54	
HbA1c (%)	7.2 (1.7)	316 (94)
Total cholesterol (mmol/L)	4.9 (1.1)	262 (78)
LDL cholesterol (mmol/L)	2.9 (1.0)	297 (88)
HDL cholesterol (mmol/L)	1.5 (0.4)	299 (89)
Serum creatinine (μmol/L)	92 (69)	338 (100)
Estimated GFR (mL/min/1.73 m^2^)	57.5 (21.5)	338 (100)
CKD stage		338 (100)
Stage 1	5	
Stage 2	45	
Stage 3	39	
Stage 4+5	11	
Diabetic retinopathy^$^	45	254 (75)
Diabetic neuropathy	84	256 (76)
Cerebrovascular disease	23	336 (99)
Coronary heart disease	21	337 (100)
Peripheral arterial disease	4	338 (100)
Diabetic nephropathy		321 (95)
Stage I	47	
Stage II	25	
Stage III	17	
Stage IV	11	
ABI	1.1 (0.1)	240 (71)
baPWV (cm/s)	1794 (397)	239 (71)
IMT (mm)	1.06 (0.27)	338 (100)

OHA: oral hypoglycemic agents, ACEi: angiotensin-converting enzyme inhibitor, ARB: angiotensin II receptor blocker, CCB: calcium channel blocker, GFR: glomerular filtration rate, CKD: chronic kidney disease, ABI: ankle-brachial index, and baPWV: brachial-ankle pulse wave velocity.

^# ^Obesity includes individuals with BMI ≥ 25 kg/m^2^.

^$ ^Diabetic retinopathy includes simple, pre-proliferative, and proliferative retinopathies.

Table 2 shows the comparison of the clinical characteristics between the patients with and without carotid IMT thickening. The frequencies of IMT thickening were significantly higher in the patients than in the normal controls (42% versus 29%, *P *= 0.02).

**Table 2:** Clinical characteristics between the diabetic patients with and without IMT thickening.

	%/Mean (SD)		
		
	IMT < 1.1 mm	IMT ≥ 1.1 mm	*P*
	(*n *= 195)	(*n *= 143)	
Age (years)	62 (12)	69 (9)	< 0.01
Men	64	65	0.78
Duration of diabetes mellitus (years)	9 (8)	13 (12)	< 0.01
Current plus past smoking	57	58	0.99
Current drinker	42	43	0.66
Therapeutic method for diabetes mellitus			
Diet only/OHA/Insulin	5/50/45	4/51/45	0.75
Body mass index (kg/m^2^)	24.7 (4.7)	24.1 (3.8)	0.19
Obesity^#^	37	34	0.53
Hypertension	73	85	< 0.01
Systolic blood pressure (mmHg)	132 (18)	133 (21)	0.40
Diastolic blood pressure (mmHg)	76 (12)	76 (12)	0.67
Antihypertensive agents			
ACEi	20	19	0.89
ARB	41	52	0.04
CCB	43	54	0.04
Hyperlipidaemia	75	71	0.38
Statin	55	54	0.85
HbA1c (%)	7.2 (1.7)	7.3 (1.7)	0.40
Total cholesterol (mmol/L)	4.9 (1.1)	5.0 (1.1)	0.29
LDL cholesterol (mmol/L)	2.9 (1.0)	3.0 (0.9)	0.25
HDL cholesterol (mmol/L)	1.5 (0.4)	1.4 (0.3)	0.31
Serum creatinine (μmol/L)	99 (81)	110 (76)	0.22
Estimated GFR (mL/min/1.73 m^2^)	61.0 (21.6)	52.8 (20.6)	< 0.01
CKD stage			< 0.01
Stage 1	7	2	
Stage 2	52	36	
Stage 3	33	48	
Stage 4+5	8	15	
Diabetic retinopathy^$^	40	50	0.096
Diabetic neuropathy	84	84	0.87
Cerebrovascular disease	19	28	0.04
Coronary heart disease	17	27	0.02
Peripheral arterial disease	4	5	0.55
Diabetic nephropathy			0.28
Stage I	49	46	
Stage II	25	24	
Stage III	19	16	
Stage IV	7	14	
ABI	1.1 (0.1)	1.1 (0.1)	0.27
baPWV (cm/s)	1736 (403)	1876 (375)	< 0.01

OHA: oral hypoglycemic agents, ACEi: angiotensin-converting enzyme inhibitor, ARB: angiotensin II receptor blocker, CCB: calcium channel blocker, GFR: glomerular filtration rate, CKD: chronic kidney disease, ABI: ankle-brachial index, and baPWV: brachial-ankle pulse wave velocity.

^# ^Obesity includes individuals with BMI ≥ 25 kg/m^2^.

^$ ^Diabetic retinopathy includes simple, pre-proliferative, and proliferative retinopathies.

The age, duration of diabetes mellitus, incidence of hypertension, and baPWV were significantly higher in the patients with IMT thickening than in those without IMT thickening. The patients receiving treatment with the angiotensin II receptor blocker (ARB) and the calcium channel blocker (CCB) were more frequent in the IMT thickening group than in the group without IMT thickening. The eGFR was significantly lower in the patients with IMT thickening than those without IMT thickening, and the incidence of IMT thickening increased with the stage progression of CKD. The prevalence of diabetic macroangiopathy manifesting as cerebrovascular disease and coronary heart disease was significantly higher in the patients with IMT thickening than in those without IMT thickening. However, there was no association between the stage of diabetic nephropathy based on the UAE and thickening of the IMT.

**Figure 1:**
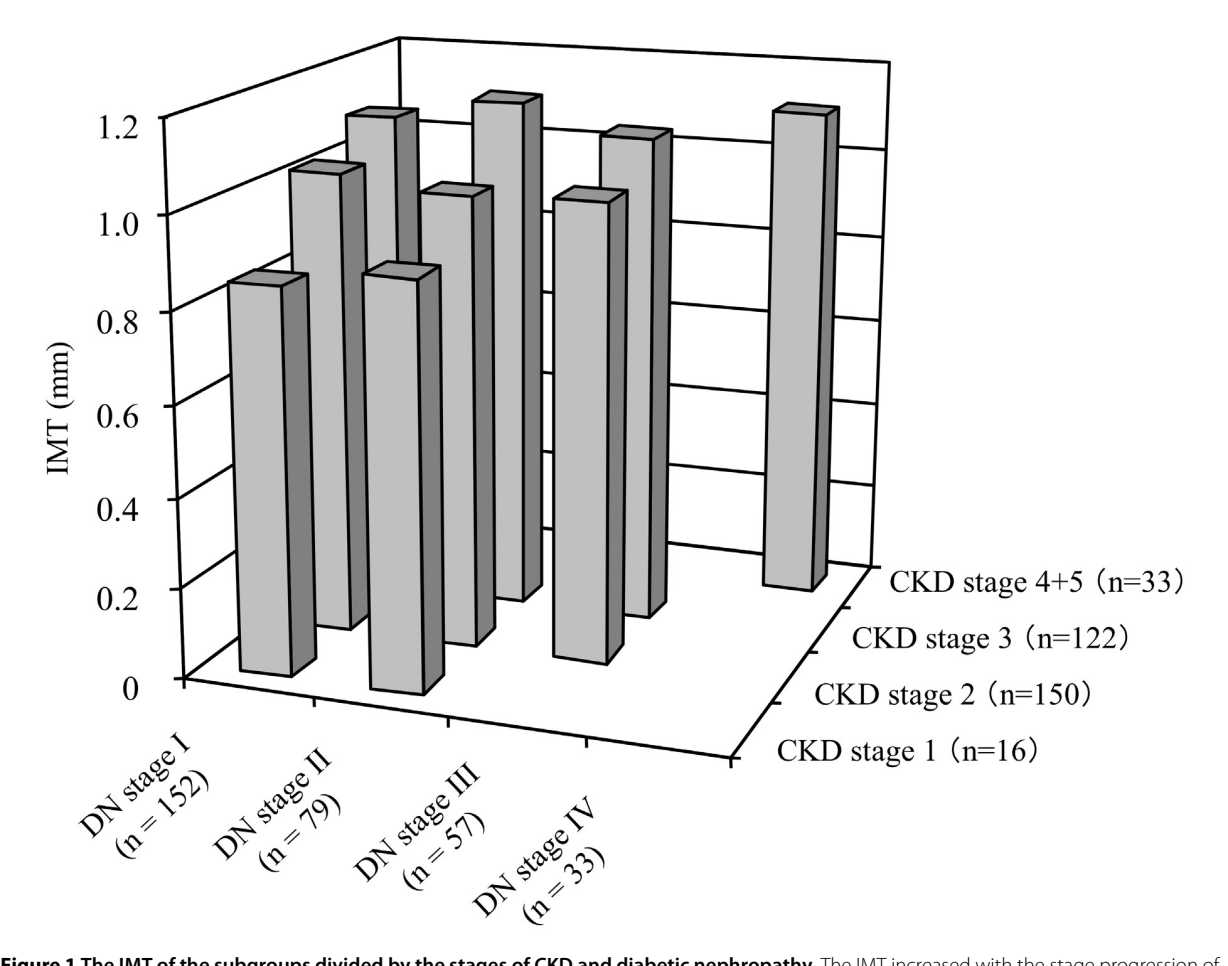
**The IMT of the subgroups divided by the stages of CKD and diabetic nephropathy**. The IMT increased with the stage progression of CKD in each stage of diabetic nephropathy (*P *= 0.03 in DN stage I and *P *= 0.01 in DN stage II).

The elevated carotid IMT significantly correlated with the stage progression of CKD (0.87 ± 0.19 mm in stage 1, 1.02 ± 0.26 mm in stage 2, 1.11 ± 0.26 mm in stage 3, and 1.11 ± 0.27 mm in stage 4+5, *P *< 0.01). However, the IMT was not significantly different among the patients at different stages of diabetic nephropathy (1.04 ± 0.27 mm in DN stage I, 1.06 ± 0.26 mm in DN stage II, 1.06 ± 0.26 mm in DN stage III, and 1.11 ± 0.28 mm in DN stage IV). Figure 1 shows the IMT among the subgroups that are divided by the stages of CKD and diabetic nephropathy. The IMT increased with the stage progression of CKD in each stage of diabetic nephropathy (*P *= 0.03 in DN stage I and *P *= 0.01 in DN stage II).

**Figure 2:**
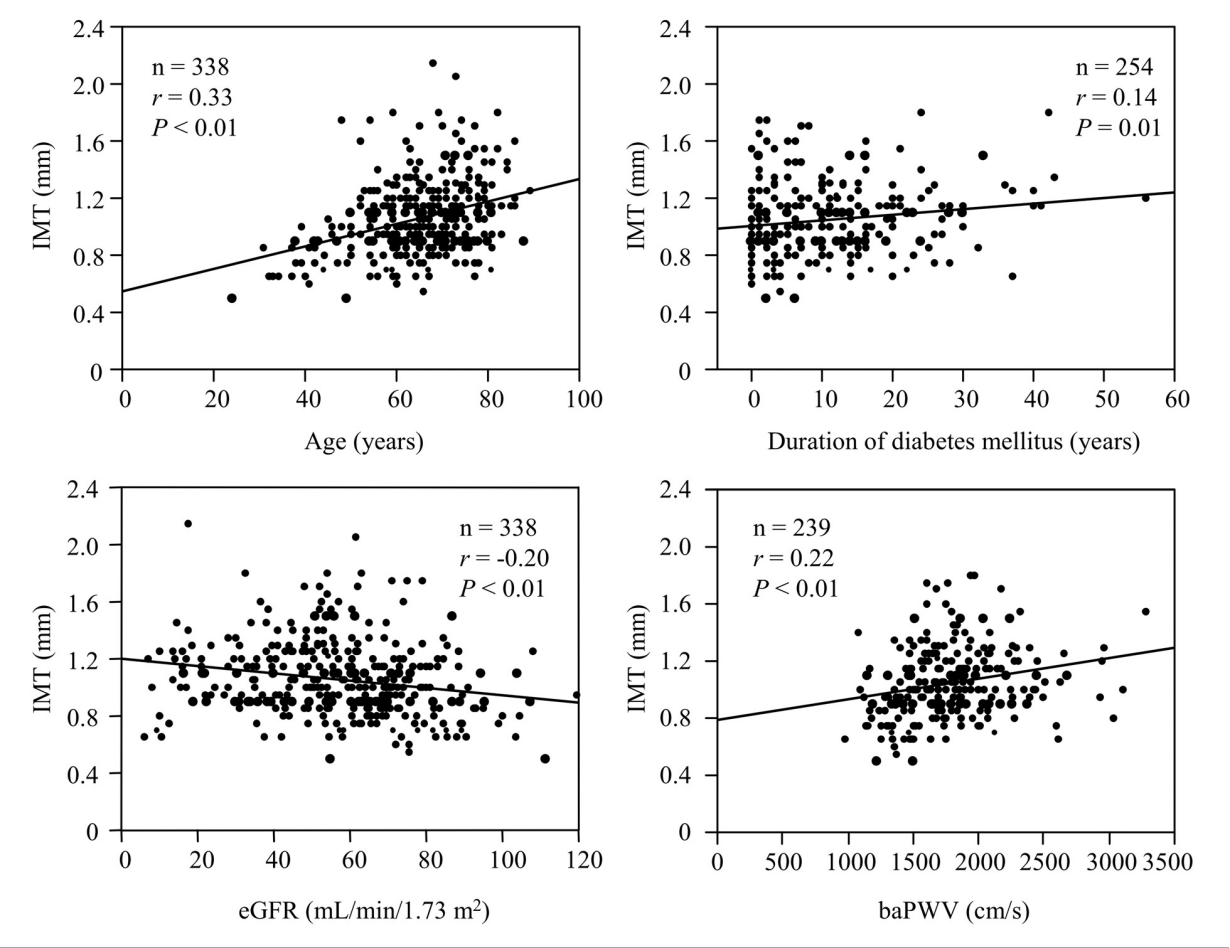
**Correlation of the IMT to the clinical parameters**. The IMT positively correlated with age, duration of diabetes mellitus, and baPWV and negatively correlated with eGFR. The level of the eGFR that the IMT was equivalent to 1.1 mm was 41.3 mL/min/1.73 m^2 ^according the formula of the correlation (y = -0.0025729x + 1.2064048).

Figure 2 shows the relationships between the carotid IMT and clinical parameters. The IMT positively correlated with age, duration of diabetes mellitus, and baPWV and negatively correlated with eGFR. The level of the eGFR that the IMT was equivalent to 1.1 mm was 41.3 mL/min/1.73 m^2 ^according the formula of the correlation (y = -0.0025729x + 1.2064048). In a stepwise multivariate regression analysis, the IMT was used a continuous variable. The following factors were entered as independent variables showing a significant correlation with IMT in the univariate analyses: the duration of diabetes mellitus, the eGFR, the baPWV, and hypertension (absent = 0, present = 1). Because the eGFR is already corrected by age, the patient's age was excluded from this analysis. The eGFR and the baPWV were independently associated with the carotid IMT (*F *= 5.53 and 6.34, *P *= 0.02 and 0.01, respectively, *r *= 0.29).

The baPWV also positively correlated with age (*r *= 0.44, *P *< 0.01) and the duration of diabetes mellitus (*r *= 0.23, *P *< 0.01) and negatively correlated with the eGFR (*r *= -0.32, *P *< 0.01). It was significantly higher in the diabetic patients with hypertension than in those without hypertension (1863 ± 394 cm/s vs. 1554 ± 301 cm/s, *P *< 0.01).

## Discussion

Based on univariate analyses in the present study, the clinical parameters associated with the IMT were age, the duration of diabetes mellitus, hypertension, the stage of CKD according to the eGFR, and the baPWV. However, the strength of the correlation was weak (*r *= -0.20) between the IMT and eGFR levels. Although the subjects with CKD (eGFR < 60 mL/min/1.73 m^2^) are considered to be high risk patients for atherosclerotic vascular diseases, diabetic patients with an eGFR < 40 mL/min/1.73 m^2 ^may have an IMT ≥ 1.1 mm according to the formula determined for the correlation. The stage of diabetic nephropathy according to the UAE was not associated with the IMT in this study. On the other hand, cerebrovascular disease and coronary heart disease were significantly more frequent in the group with IMT thickening than that without IMT thickening. These results are in accordance with the use of the IMT as a surrogate marker of atherosclerotic diseases.

Agewell *et al*. reported a significant association between the carotid IMT and the UAE in 368 healthy 58-year old males [19]. In the random population which included diabetic patients, the carotid IMT was also related to the UAE, although the evaluation of renal functions, such as the GFR was not investigated [20,21]. Kawamoto *et al*. demonstrated a negative correlation between the carotid IMT and the eGFR without an examination for the UAE [22]. Hermans *et al*. showed that the carotid IMT was significantly associated with both the UAE and the eGFR in 806 subjects, including 181 subjects with impaired glucose tolerance and 326 with type 2 diabetes mellitus.

However, the significance disappeared after an adjustment by the other parameters, such as subject's age [23]. Preston *et al*. reported that the carotid IMT increased with the attenuation of the eGFR in 104 CKD patients, including 33 diabetic patients, with a serum creatinine level > 125 μmol/L. They also did not find an association between the IMT and the UAE [24].

Several studies were performed to investigate the association between the IMT and the clinical parameters in the patients with type 2 diabetes mellitus [12-17,25]. Taniwaki *et al*. reported that the IMT was not different between the groups with normoalbuminuria and microalbuminuria but that it was negatively correlated with the eGFR in 61 patients with type 2 diabetes mellitus [12]. Yokoyama *et al*. and Keech *et al*. demonstrated that IMT elevated with an increase in the UAE [13,14], although Mykkänen *et al*. and Ishimura *et al*. reported that the IMT did not correlate with UAE [9,15]. Dong *et al*. observed a significant association between the IMT and the eGFR in 1,009 Chinese diabetic patients without an evaluation of the UAE [16]. Freedman *et al*. reported that carotid IMT did not differ among the groups with normoalbuminuria, microalbuminuria, and macroalbuminuria in 588 white participants with type 2 diabetes mellitus [17]. Our results also indicate that the IMT negatively correlated with the eGFR. Moreover, our study is the first report showing a relationship between the carotid IMT and the renal parameters including eGFR and the stages of diabetic nephropathy with a confirmed association between the IMT and macroangiopathy. Although previous studies have not refuted a relationship between the IMT and the GFR, an association between the IMT and the stage of diabetic nephropathy based on the UAE showed a discrepancy. This might be explained by the differences in the patients' background, such as age and duration of the illness. Furthermore, it is possible that individuals with an increased UAE and IMT died prior to inclusion into this study. Recently, it was reported that both the decreased eGFR and proteinuria are independently associated with cardiovascular events in both the random population [26] and in the patients with type 2 diabetes mellitus [27]. Our results and the discrepancies in the previous reports might be explained if the effect on cardiovascular events is stronger in the subjects with the decreased eGFR than in those with proteinuria. However, further studies will be needed to confirm this.

It was recently reported that the IMT was significantly associated with diabetic neuropathy [25]. The IMT was not different between the patients with and without diabetic microangiopathy, including diabetic neuropathy in this study. The older age and the high frequency of diabetic neuropathy of the patients in this study might be the reason for the different result.

In this study, the patients receiving treatment with the ARB and the CCB were frequent in the group with IMT thickening compared to the group without IMT thickening. This indicates that more antihypertensive agents were necessary in the group with IMT thickening to reduce blood pressure because of the similar systolic and diastolic blood pressure between the two groups (Table 2).

The beneficial effects of the renin-angiotensin system (RAS) inhibitors for renal dysfunction and vascular adverse events are widely recognized. It was reported that telmisartan, one of the ARBs, reduced the IMT compared to amlodipine in the hypertensive patients with CKD [28]. The ARB might be useful in the diabetic patients with both an IMT thickening and a decreased eGFR for the prevention of vascular events as well as a renoprotective effect. Other investigators reported that amlodipine also decreased the IMT in the hypertensive patients [29,30]. Furthermore, Ikeda *et al*. reported that amlodopine significantly decreased the IMT compared to losartan in 104 hypertensive patients with type 2 diabetes [31]. It is unclear whether this discrepancy is caused by the different backgrounds of the study subjects and whether the different effects are observed among the ARBs. Statin was also reported to reduce the IMT in the patients with CKD, including diabetes mellitus [32]. In this study, the effects of the antihypertensive agents and statin for the prevention of diabetic complications were not able to be evaluated because our study is based on a cross-sectional study with the patients receiving the treatments prior to the study.

The baPWV is another measure of atherosclerosis. While the IMT provides a measure of the arterial thickening, baPWV reflects arterial stiffness and is a marker of both the severity of vascular damage and the prognosis of atherosclerotic disease in the patients with diabetes mellitus [32]. The IMT showed a significant positive correlation with the baPWV in our study. This finding is in accordance with previous studies [6,13,15]. Both an IMT thickening and an increased baPWV are likely to reflect the early stages of atherosclerosis. Because the eGFR was associated with both the IMT and the baPWV in this study, the eGFR might be another useful surrogate marker for vascular events in the patients with diabetes mellitus.

Yamashina *et al*. reported that a baPWV > 1400 cm/s is an independent risk factor for atherosclerotic cardiovascular disease [33]. Because the baPWV levels were high in both groups with and without IMT thickening, the study subjects of this study were considered to be at an increased risk for vascular events. However, the mean IMT was within the normal range in this study. The discrepancy in the level of risk and IMT might have been caused by the factors which influence the baPWV, such as arterial calcification and hypertension.

The results of this study have several limitations that must be considered. First, the findings are inherently limited by an inability to eliminate causal relationships between the risk factors and carotid IMT thickening because of the cross-sectional design. Although cerebrovascular and coronary heart diseases were frequent in the patients with carotid IMT thickening, this study did not examine diabetic macroangiopathies as the direct vascular endpoints. The effects of drugs, such as antihypetensive agents and statin, were also not able to be evaluated. Therefore, our data can not provide the mechanisms for the observed associations between the clinical parameters and the IMT. It should be noted that our results are unable to explain the potential confounding effects of the RAS inhibitors and statin on the arterial remodeling process. Second, the evaluations of the eGFR, the UAE and the IMT were performed according to a single assessment of the serum creatinine level, the urinary albumin concentration, and the carotid artery ultrasonography. We also have to be mindful of to the possibility of either misclassification or bias in the staging of diabetic nephropathy and CKD. Third, there were some missing data with regard to the duration of diabetes mellitus, smoking history and baPWV. It is also possible that an error could have occurred during the multivariate analysis.

In conclusion, intensive examinations to improve the early detection of atherosclerosis, and earlier treatments for hypertension, dyslipidaemia, and obesity as well as hyperglycaemia are necessary to prevent future cardiovascular adverse events when a reduced eGFR related with IMT thickening is found in the patients with type 2 diabetes mellitus. However, further studies investigating the clinical outcomes are needed because this study is based on a cross-sectional design.

## Competing interests

The authors declare that they have no competing interests.

## Authors' contributions

HI contributed to design of the study, analysis and interpretation of data, and drafting of the manuscript. YK contributed to measure the IMT with ultrasonographic examinations. MM, AS, HI, TY, HI and MT contributed to interpretation of data and critical revision of the manuscript. All authors have given the final approval of the version of the manuscript to be published.
